# Hydrogel-driven solar desalination: integrating material innovation with interfacial water transport

**DOI:** 10.1039/d6ra04617g

**Published:** 2026-07-21

**Authors:** Tholkappiyan Ramachandran, Bathina Chaitanya, Ramesh Kumar Raji, Vadivelan Subramaniyan, Shanmugavel Chinnathambi, Ganesan Subramanian, Karthishwaran Kandhan, Renuka Seenivasan, Fathalla Hamed

**Affiliations:** a Department of Physics, College of Science, United Arab Emirates University P. O. Box 15551, Al-Ain Abu Dhabi United Arab Emirates thols2006@gmail.com shanmugavelc.phy@citchennai.net; b Amrita School of Computing, Amrita Vishwa Vidyapeetham, Amaravati Campus Kuragallu, P. O. Box 522240 Andhra Pradesh India; c Karpaga Vinayaga College of Engineering and Technology, Chinnakolambakkam P. O. Box 603308 Chengalpattu Tamil Nadu India; d Centre for Applied Nanomaterials, Chennai Institute of Technology Kundrathur Chennai–600 069 India; e Department of Computer Studies & Engineering, Symbiosis International University Dubai Dubai Knowledge Park Dubai P. O. Box 502930 United Arab Emirates; f Department of Computer Science & Engineering, Symbiosis International (Deemed University) Pune P. O. Box 412115 India; g Department of Integrative Agriculture, College of Agriculture and Veterinary Medicine, United Arab Emirates University P. O. Box 15551 Al Ain United Arab Emirates; h Engineering Technology and Science, Higher Colleges of Technology Abu Dhabi Baniyas Campus, P. O. Box 60074310 United Arab Emirates

## Abstract

In recent years, new materials based on hydrogels have been identified as innovative platforms for solar desalination, offering novel possibilities for the integration of water transport, light harvesting and salt management in a single system. As hydrogels do not require a solid evaporator structure, they offer the inherent benefit of enabling the continuous delivery and adjustment of water, which can be finely controlled by controlling the transport, as opposed to the traditional rigid evaporator structure. The review covers the physical and chemical bases of the hydrogel-based desalination process in detail with emphasis on water, heat and ion transport mechanisms. In addition to the significance of polymer–water interactions and various forms of confined water in evaporation control, recent advances in the understanding of salt transport and evaporation entropy effects are discussed in detail. With a mechanistic perspective based on thermodynamics and transport principles, this review elucidates the principles that link the material structure and physicochemistry of hydrogels to their desalination properties. Hydrogel structures, ranging from layer-by-layer constructs to bioinspired constructs, are introduced, and the challenges associated with material performance in solar desalination applications are addressed. Moreover, discrepancies among the reported material performances and guidelines for performance evaluation are discussed. Overall, this review aims to provide guidance for the development of future solar desalination strategies based on hydrogel structures.

## Abstract 2

Hydrogel-enabled solar desalination: coupling water transport, ion regulation, and salt management for sustainable freshwater production.

## Graphical abstract

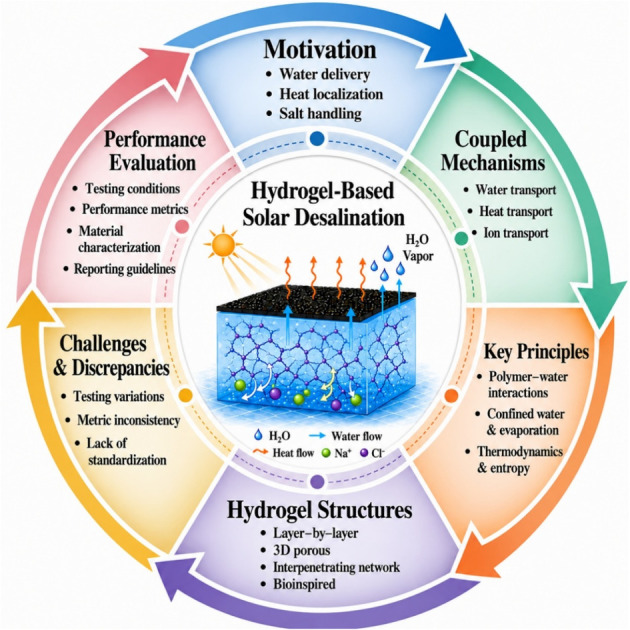

## 1. Introduction

In the twenty-first century, one of the most pressing challenges has been freshwater scarcity caused by population growth, increased industrial activities, changing climates, and irregular geographical distribution of water sources.^1–3^ Water covers approximately 71% of the surface area of Earth, but less than 2.5% corresponds to freshwater, of which only a small part can be used by humans and for agricultural purposes.^4^ Water stress is a critical issue in areas of desert and semi-desert landscapes, where population growth and development create a demand for water. According to the projections, in the coming decades, billions of people will be living in extreme water scarcity scenarios; therefore, sustainable and energy-efficient solutions that can be implemented on a decentralized basis are required.^5^ A potential method is the use of seawater and brackish water desalination, which can yield an unlimited quantity of fresh water. Solutions based on reverse osmosis and thermal technologies, such as multi-stage flash distillation and multi-effect distillation, have reached a high level of technological maturity but are all energy- and energy-plant-intensive and based on centralized installations and centralized fossil-fuel energy production. Furthermore, they suffer from greenhouse gas emissions and discharge of concentrated brine, which restrict their long-term sustainability and scalability.^6–8^ It is therefore important to find alternative desalination technologies for seawater and brackish water that can be operated in an energy-sustainable, low-infrastructure and friendly manner.

Some of the techniques employed include solar-driven desalination technologies that have attracted significant attention because they have direct access to unlimited solar energy. Interfacial solar evaporation technology is a promising concept that can be implemented in an energy-sustainable way. Since interfacial devices confine all heat generation processes to the air–water interface, they allow for the minimization of thermal losses and the maximization of the localization of evaporation.^9^ It means that interfacial desalination provides high solar-to-vapor conversion efficiency and does not require any external energy supply; thus, it represents a particularly attractive concept for decentralized freshwater production. Further developments in photothermal materials have led to improvements in photothermal conversion efficiency, including introduction of carbon-based materials, plasmonic materials, and semiconductors.^10^ As a result, novel material platforms that integrate different functions of evaporation interfaces have been created. Hydrogels have been rapidly developing into one of the most attractive types of materials for interfacial solar evaporation. A hydrogel is a three-dimensional cross-linked polymer network containing a significant amount of water in their matrix; hence, they have the properties of liquids and solids. The high-water absorption, capillary pressure and ability to build wetting forces mean that hydrogels can allow water to flow continuously from a bulk solution to an evaporator interface without the use of any additional pumping. This contrasts with rigid evaporator structures which require other pumping systems. Importantly, porosity, wettability and water transport are being controlled using polymer chemistry, cross-linking and modification of functional groups.^11^

Hydrogels are a promising material platform as they can make the passive water absorption-based solar desalination process a dynamic material phenomenon involving water/fuel, heat and ion transport. The high water content allows for tunable interactions between the polymeric network and water molecules, which, in turn, allow for the production of different types of water content (free, bound and intermediate) with different effects on the evaporation kinetic and evaporation energy.^12^ At the same time, photothermal additives can create highly efficient light absorbers within hydrogel networks; thus, heat localization in these materials can be optimized. Besides water transport, hydrogels possess chemical functionality that allows for the interaction of ions with polymers. Specifically, certain functional groups can lead to selective ion–polymer interaction, resulting in Donnan effects, ion affinities, and delays in the crystallization of precipitates.^13^ These features represent important ways to prevent salt accumulation during the evaporation of saline water. In addition, unlike rigid materials, hydrogels can endure stress caused by salt crystallization due to their elasticity. The above-mentioned features of hydrogels along with their bio-inspired softness and high-water content enable the simulation of biological water–evaporation processes, for example, transpiration of plants. Therefore, hydrogels are being developed in the direction of designing increasingly complex systems in which light harvesting, water transport, and salt management are integrated into a single material platform.^14^

**Fig. 1:**
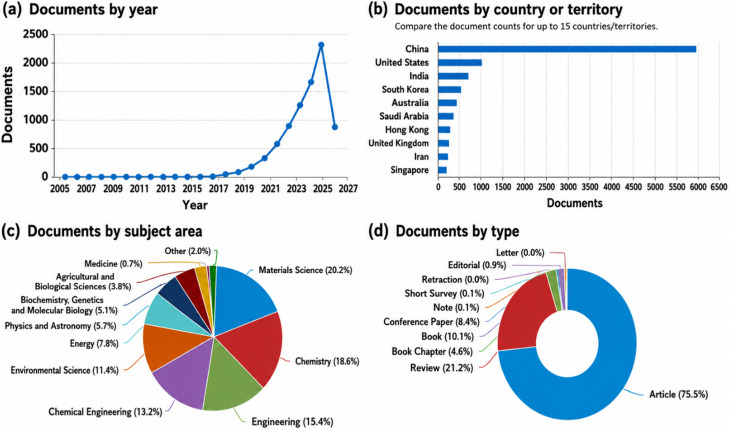
Bibliometric analysis of the hydrogel-based solar desalination research area: (a) publication trends over the years, (b) geographical location of the publications, (c) distribution by the subject area and (d) distribution of the publication types.

However, a limited number of studies have addressed the physics and chemistry underlying hydrogels' water evaporation mechanisms in sunlight. Indeed, much of the existing research focuses on material composition, additives to enhance the photothermal efficiency, and record-high evaporation rates; however, in doing so, hydrogels are seen as passive materials rather than as actively participating entities in the evaporation process.^15^ As a result, many aspects of the physics and chemistry of water evaporation and salt transport processes in hydrogels are not yet studied. There remain many important unresolved questions about the nature of polymer–water–ion interactions, reduction in evaporation enthalpy, and unusually high solar conversion efficiency. Importantly, water evaporation at the air–water interface is a non-equilibrium process, which makes it difficult to use the principles of classical thermodynamics. At the same time, hydrogels exhibit highly coupled multiphysics phenomena such as heat transfer, mass transport, and ion transport. Such multiphysics interactions take place on a multiscale level, including molecular interactions, mesoscale network structure, and macroscale geometry of hydrogels.^16^ For example, enhanced water transport might result in higher thermal losses; at the same time, limiting the ion diffusion can cause salt accumulation in the polymer matrix. However, existing research lacks an in-depth study on structure–property–performance relationships, which is needed. Besides fundamental research, some challenges need to be addressed to enable practical applications that depend on the desalination process based on hydrogels. These comprise long-term stability, biofouling and salt fouling resistance and scalability of hydrogel production.^17–20^ Hydrogel-based solar desalination technology trends and directions were illustrated by means of bibliometric analysis, as shown in Fig. 1. The bar chart in panel (a) shows that these publications were largely unaffected until 2015 when they started to increase according to an exponential curve. The pie chart in panel (b) presents the geographical distribution of publications. China ranks first by its contribution to hydrogel-based desalination with a considerable margin; the next largest contributors are the USA, India, and South Korea. This trend reflects investments of countries experiencing problems related to water scarcity and environmental pollution into the development of innovative technologies.^21^ The chart in panel (c) demonstrates the disciplinary distribution of hydrogel-based solar desalination research. The largest share corresponds to materials science, which reflects the central focus of research on designing new materials and using photothermal substances. Contributions from the domains of chemistry and engineering indicate the important roles of reaction mechanisms and polymer synthesis; besides, the presence of research in environmental sciences and energy-related areas demonstrates the applied orientation of this research field.^22^ The pie chart in panel (d) indicates that the primary format of research publication is research articles, followed by review articles, which reflect both active scientific progress and the necessity of summarizing accumulated knowledge.^23–27^

**Fig. 2:**
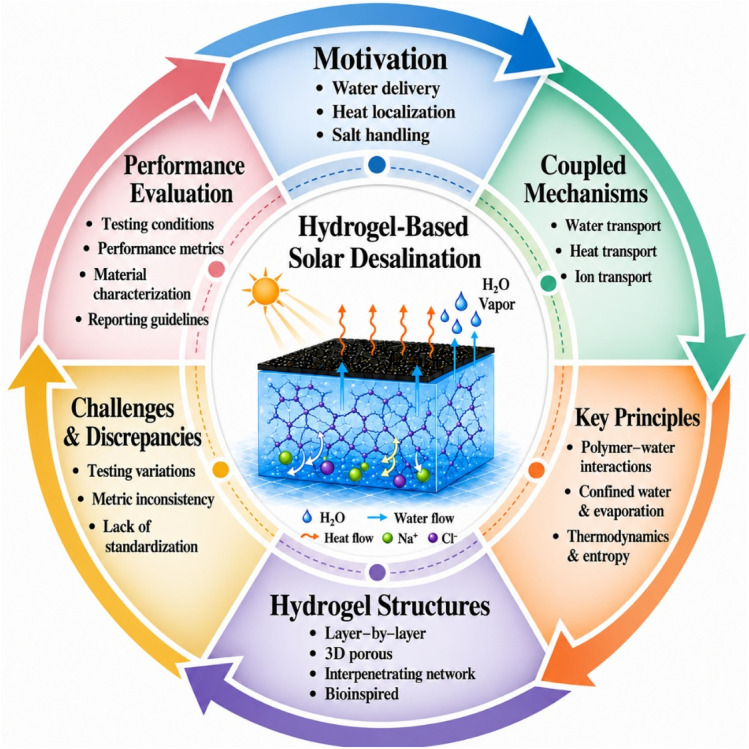
Schematic of the hydrogel-based solar desalination processes.

In this context, the present review will attempt to give a comprehensive mechanism- and material-centric review of the desalination based on hydrogels. The present paper focuses on the principles that underline the functionality of hydrogels, which are used upon irradiation with sunlight rather than giving a description of the materials. In particular, the importance of the interfacial water, the polymer-induced ion transport and the thermodynamics of evaporation will be extensively studied, in addition to the most recent advances regarding the entropy and confinement phenomena. This paper attempts to fill these gaps in literature by critically examining the methodologies used for the experiments, efficiency measurement, and underlying assumptions, to build a solid understanding of the evaporation processes of the hydrogel. The layout of this review is structured such that gradual progression from fundamentals to applications will be achieved. Hydrogels physicochemical properties are going to be discussed first. Second, interfacial water and salt transport processes will be given in detail, which is the basis for the understanding of the evaporation mechanism of hydrogels. Further discussions will address thermodynamic principles, design principles for hydrogels and methods to enhance stability and to address fouling problems. Scalability, sustainability, system integration, standardized and benchmarking hydrogel desalination systems will then be discussed. In conclusion, future directions in terms of scientific and engineering challenges will be summarized. The current review will show the multi-disciplinary aspects of solar desalination using hydrogels, based on the knowledge obtained from polymer science, interfacial physics, transport science and environmental engineering. The pillars of hydrogel-based solar desalination systems are fundamentals, transport processes, thermodynamic principles, architectures, stability and fouling mitigation, scalability, sustainability, and standardization and benchmarking, as demonstrated in Fig. 2.

## 2. Fundamentals of hydrogel materials for solar evaporation

**Fig. 3:**
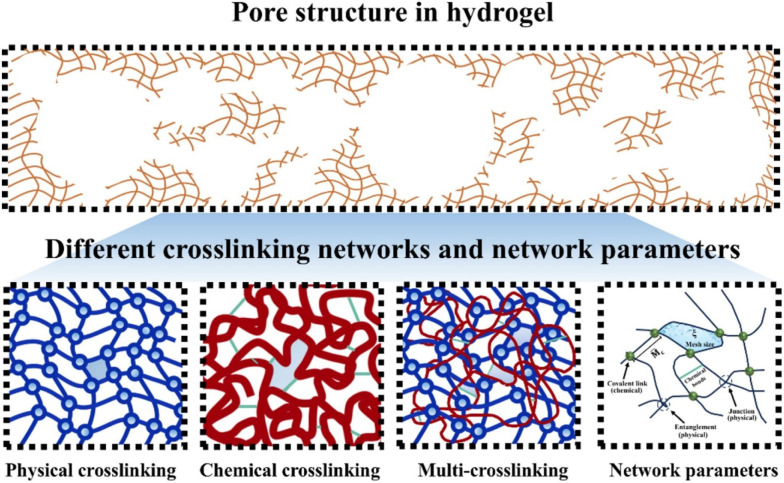
Polymer network design and pore structure in hydrogels. Reproduced from ref. 32 with permission from Elsevier, copyright 2025.

One of the key applications of hydrogels is to retain a significant amount of water in their polymeric structures. In the solar powered desalination process, hydrogels are used as water reservoirs and as active phases, which can regulate the light absorption, heat trapping, and mass transportation processes at the interface of water evaporation.^28^ The unique properties of hydrogels are its intrinsic combined flexibility and a high-water content, which can continuously supply water to the evaporation surface without causing much heat transfer from the heated layer to the rest of water. Unlike inflexible photothermal materials, hydrogels have unique characteristics, a combination of flexibility with the presence of abundant water, which can continuously supply water to the evaporation area without significant heat transfer from the heated layer to the remaining water body.^29^ These benefits render hydrogels particularly attractive for the case of interfacial solar evaporation system in which ideal control of energy and water exchange at the evaporation surface is necessary for efficient desalination.^30^ The performance of hydrogel-based evaporators depends on several factors related to polymer chemistry, hydrogel structure, and physical interactions between the polymer framework and water and its ions. The crosslinking degree, polymer hydrophilicity, and hydrogel pore structure have impacts on water state, distribution, and dynamics in the network.^31^ Additionally, hydrogels offer the possibility of loading various photothermal materials, such as carbon-based composites, plasmonic nanoparticles, and conjugated polymers through physical incorporation or chemical immobilization.^32^ Thus, the decoupling of light absorption and water transport can be achieved using hydrogel-based platforms in an easier manner than for other evaporator types. As far as the mechanism of action of hydrogels in solar evaporation is concerned, the peculiarities of water confined in polymeric structures should be considered. Due to specific hydrogen-bonding configurations, different heat response, and altered mass transport processes, such water demonstrates features distinct from bulk water that cannot be described by traditional models of evaporation. Understanding the underlying processes associated with light-to-energy conversion and material–water interactions is key to rationalizing the results obtained in the field of hydrogel-based solar desalination.^32^ Therefore, knowledge about the physical–chemical properties of hydrogels plays a crucial role in designing efficient solar-powered evaporators in the future. Fig. 3 highlights how the polymer network structure determines the pores in the hydrogel and the states of water in the pores.

### 2.1 Polymer networks and crosslinking strategies

The backbone of hydrogels lies in the polymer network, whose design depends on the inherent nature of the polymers themselves, and the crosslinking approaches utilized to connect them. Hydrogels chemically crosslinked through covalent interactions offer exceptional stability and resistance to dissolution, making them appropriate candidates for desalination processes requiring extended exposure to sunlight.^33^ Some common crosslinking techniques are free-radical polymerization, click chemistry, and photoinitiated crosslinking, providing excellent control over network density and mechanical strength. Yet, an excessive degree of crosslinking restricts the movement of water molecules within the network, hindering both evaporation and ion removal processes. Alternatively, physically crosslinked hydrogels depend on reversible bonds, including hydrogen bonding, ion pairs, crystallites, or hydrophobic complexes.^34^ In physical hydrogels, the network structure changes reversibly depending on the changes of environmental parameters such as salinity, temperature or hydration during desalination. Additionally, when physical crosslinking occurs, it adds to self-healing properties and increases tolerance to mechanical pulling or pushing from salt crystallites. The negative aspect of physical hydrogels is, however, their lesser mechanical stability and durability under adverse environmental conditions or in cycles of swelling and drying.^35^ There are some recent developments that pay attention to a mixture of chemical and physical crosslinking. Double-network hydrogels, supramolecular systems, and ionic hydrogels have been found to exhibit greater mechanical strength without adversely affecting the water permeability. In terms of solar desalination applications, these inventions enable mechanical strength to be separated from transport capabilities, thus enabling continuous operation of hydrogels and maintaining evaporation performance. Therefore, it becomes crucial to comprehend the relationships between crosslinking chemistry, network dynamics, and water transport to develop effective hydrogel materials for solar desalination applications.^36^Table 1 lists the structure–property–function relationships of hydrogels for solar evaporation.

**Table 1:** Structure–property–function relationships of hydrogels for solar evaporation

Hydrogel feature	Design parameter/example	Dominant water state	Optical/thermal behavior	Impact on solar evaporation	Reference
High crosslink density	Covalently crosslinked PAAm and PVA	Bound and intermediate water	Low thermal conductivity and strong heat localization	Efficient interfacial heating but limited water replenishment	37
Low crosslink density	Physically crosslinked hydrogels	Free water dominant	Higher thermal conductivity	Fast water transport but increased heat loss	38
Dynamic crosslinks	H-bonded, ionic, and supramolecular	Dynamic exchange between water states	Adaptive thermal response	Improved salt tolerance and long-term stability	39
Double-network hydrogels	Covalent + physical networks	Mixed water states	Balanced heat and mass transport	High evaporation rate with good mechanical durability	40
Hydrophilic functional groups	–OH, –COOH, and –SO_3_^−^	Increased bound/intermediate water	Altered hydrogen-bonding network	Reduced effective evaporation enthalpy (reported)	41
Zwitterionic polymers	Sulfobetaine and carboxybetaine	Strongly bound hydration layer	Stable thermal behavior	Excellent antifouling and salt resistance	42
Large pore size	Macroporous hydrogels	Free water	Weak thermal insulation	High evaporation flux and low efficiency	43
Hierarchical porosity	Micro–meso–macro pores	Coexisting water states	Controlled heat localization	Optimized evaporation efficiency	44
High water content	>90 wt% water	Free + intermediate water	Enhanced heat dissipation	Increased flux but lower solar efficiency	45
Reinforced hydrogel matrix	Fiber- or mesh-reinforced	Mixed water states	Stable thermal pathways	Improved mechanical robustness for long-term operation	46
Surface-modified hydrogels	Janus and hydrophobic top layer	Free water at interface	Reduced convective heat loss	Enhanced solar-to-vapor efficiency	47
Bio-derived polymers	Cellulose, alginate, and chitosan	Bound/intermediate water	Moderate thermal insulation	Sustainable evaporation with moderate performance	48

### 2.2 Water states in hydrogels

**Fig. 4:**
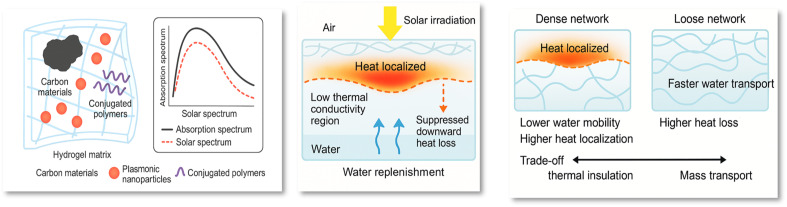
Photothermal integration and thermal management interacting with the hydrogel structure to localize heat and regulate the evaporation efficiency.

The water that is held within hydrogel network structures does not behave like water in bulk. Rather, it can exist in several physico–chemical states formed through the effects of interactions with polymer chains and hydrogel network structures. This has led to the classification of such water states as being free water, intermediate water, and bound water.^49^ Free water exists in the bigger pores and/or voids in the hydrogel structures and behaves like bulk water because it can contribute significantly to evaporation. On the other hand, bound water exists close to hydrophilic functional groups in the polymer chains with very strong hydrogen bonding or even ionic interactions, which lead to restricted movement and inhibition of phase transition behavior. Intermediate water exists in an environment with partially interacting polymer chains but can still retain some movement.^50^ The types of water that can form in hydrogels can depend on the hydrogel's chemical composition and structure. For instance, a greater tendency towards having hydrophilic functional groups in the polymer chains (hydroxyl, carboxyl, sulfonate, *etc.*) leads to greater amounts of bound and intermediate water. On the other hand, loosely crosslinked structures with large pores lead to the prevalence of free water.^51^ Other parameters affecting the water state behavior in hydrogels include crosslink density, polymer chain rigidity, and ion content. Various experimental techniques including differential scanning calorimetry (DSC), nuclear magnetic resonance (NMR) spectroscopy, and Fourier transform infrared (FTIR) spectroscopy have been widely used to study water states in hydrogels and to determine the amounts of bound water and free water.^52^Fig. 4 shows the role played by the integration of photothermal heating and thermally induced regulation of hydrogel structures in localizing heat for evaporation enhancement. The existence of different water states in a hydrogel can have significant impacts on solar energy-induced evaporation and desalination. Bound and intermediate water tend to exhibit lower evaporation enthalpy values due to the disruption of hydrogen bonding in them. This phenomenon has been cited as the reason for low latent heats and high evaporation efficiencies seen in hydrogels.^53^ While the contribution of bound water in vapor generation has been questioned since they have low mobility, it is probably in the water exchange process from solar heating where water states can play a major role. Bound and intermediate water may be released into more mobile free water through a change in water states caused by photothermal heating. Understanding the mechanisms of water-state transformation in photothermal evaporation becomes critically important in achieving higher efficiencies and in designing hydrogels for efficient solar desalination.^54^

### 2.3 Optical and thermal properties

Effective desalination through solar energy relies heavily on the capability of the evaporator to absorb the received sunlight efficiently, and at the same time, concentrate heat around the air–water boundary. Pure hydrogels are mostly transparent and have poor light absorption characteristics in the solar spectrum range; therefore, photothermal elements are necessary for solar energy capture. A diverse range of materials such as carbon nanomaterials, plasmonic nanoparticles, semiconductors, and conjugated polymers have been added to hydrogels for broadband light absorption.^55^ Efficient light absorption requires that the light-absorbing particles be homogeneously dispersed and stabilized in the polymer matrix. In addition to their role in absorbing the incoming solar energy, the thermal conductivity of hydrogels determines the efficiency of desalination through solar energy. Hydrogels, by virtue of being composed of porous structures with a relatively large amount of water, do not facilitate efficient heat conduction. Consequently, less heat will be transferred to the underlying bulk liquid water, leading to heat concentration, and hence, increased efficiency at the air–water boundary under solar energy.^56^ Thermal conductivity depends on parameters such as network architecture, water content, and filler materials. The network should have proper thermophysical properties to balance both thermal insulation and effective liquid transport. If the insulation is too high, water replenishment will be inhibited, and if it is too conductive, it will cause heat dissipation. Table 2 lists the photothermal materials integrated into hydrogels for solar evaporation.

**Table 2:** Photothermal materials integrated into hydrogels for solar evaporation

Photothermal material type	Typical examples	Absorption characteristics	Integration strategy	Key advantages	Limitations	Representative reference
Carbon-based materials	CNTs, graphene, and carbon black	Broadband (UV-vis-NIR)	Physical embedding and coating	Low cost and strong absorption	Aggregation and fouling risk	57
Plasmonic nanoparticles	Au, Ag, and Cu	Narrow and LSPR-based	*In situ* growth and dispersion	High local heating	Cost and stability	58
Conjugated polymers	Polypyrrole and polyaniline	Tunable broadband	Copolymerization	Flexible and lightweight	Photodegradation	59
MXenes	Ti_3_C_2_T_*x*_	Broadband, strong NIR	Composite formation	High photothermal efficiency	Oxidation sensitivity	60
Transition metal sulfides	MoS_2_ and WS_2_	Broad visible absorption	Embedded nanosheets	High efficiency	Processing complexity	61
Hybrid fillers	Carbon + plasmonic	Synergistic absorption	Multicomponent hydrogels	Improved robustness	Fabrication complexity	62
Bio-inspired absorbers	Melanin and polydopamine	Broadband	*In situ* polymerization	Biocompatibility	Moderate efficiency	63

Optical absorption and thermal management play important roles in hydrogel-based evaporators, since photothermal effects occur in a hydrated environment that changes dynamically. Local heating may induce local temperature gradients, which affect polymer chain motions, water diffusion, and ion migration in hydrogels.^64^ Moreover, photothermal materials may affect optical properties and mechanical and hydrophilic properties of hydrogels as well, resulting in new trade-offs. Therefore, it is necessary to develop the theory of how optical absorption, heat dissipation, and heat transfer are related to polymers and water. This is required for high efficiencies of solar-to-vapor energy conversion and desalination.^65,69^

## 3. Hydrogels interfacial water, heat, and salt transport mechanisms and non-equilibrium thermodynamics

Hydrogel-based solar desalination technology performance depends critically on the joint transport phenomena of water, heat, and salt ions within an interacting polymer network subject to solar non-equilibrium heating. Unlike porous or rigid materials used as evaporation devices, hydrogels present a dynamic environment where molecular interactions, the hierarchical network topology, and external influences jointly determine the transport mechanisms.^66^ Evaporation in hydrogels thus cannot be fully explained with classical concepts relying exclusively on bulk diffusion and capillary flow principles; rather, it is a result of interactions between polymer and water molecules, transport pathways, local temperature gradients, and thermodynamic forces. Quantitatively, this can be described through transport equations coupled with interfacial thermodynamics, where material properties including crosslink density, porosity, and functional groups play a direct role in setting the transport coefficients and energy transfer pathways.^67^ Specifically, hydrogel evaporation needs to be described in terms of nonequilibrium thermodynamics where energy input, mass transport, and entropy generation processes are intrinsically coupled. For this reason, the transport processes are discussed in conjunction with the thermodynamics analysis, resulting in the establishment of structure-transport-thermodynamics-performance relations.

### 3.1 Hydrogel network water transport pathways

Adequate supply of fresh water to the evaporation interfaces is a key aspect of efficient solar desalination. In hydrogel systems, water transport involves capillary-driven flow, diffusion across the polymer network, and the evaporation-driven migration, each depending greatly on the network architecture.^68^ On the mesoscopic scale, hydrogels can be considered as porous structures where water exists within the voids and polymer-bound regions. Capillary-driven transport dominates in the cases of structured pores or channels, whereby the capillary pressure gradient enables fast transport upwards towards the evaporation interface. It can be described using the Lucas–Washburn equation:*L*^2^ = (*γr*cos *θ*/2*µ*)*t*,where *L* stands for the transport length, *r* the channel or pore radius, *γ* the surface tension, and *µ* the fluid viscosity. Capillarity-driven water transport is characteristic of CNF-based hydrogels, wood-like anisotropic structures, and freeze-casted hydrogels like PVA. In hydrogels with no specific pores/channels, water transport occurs *via* diffusion through the polymer network. This process is ruled by Fick's law, stating that the water flux is proportional to its concentration gradient:*J*_w_ = −*D*_eff_∇*C*,where the effective diffusivity (*D*_eff_) is typically described as *D*_eff_ = *D*_0_(*ε*/*τ*), with *ε* being the porosity and *τ* the tortuosity of the network. Increasing the crosslink density of homogeneous polymer hydrogels like PAAm and PVA results in smaller mesh size and thus decreases the effective diffusivity.^70^ An important parameter characterizing the transport pathways in the hydrogel network is the network tortuosity, which measures the degree of complexness in the transport pathways. As tortuosity increases, it leads to reduced effective permeability, and thus, can cause heterogeneous water distribution, especially when unevenly heated. Similarly, the water transport regime is also determined by the pore/channel size and their distribution across the network. Importantly, water transport in the hydrogels is inherently tied to evaporation-driven mass transport. The evaporation flux can be formulated as *J*_evap_ = *m*/*A* and should meet the condition *J*_evap_. At the same time, water transport is intimately related to energy balance: *ṁ* = *Q*_in_ − *Q*_loss_/*hlv*, meaning that the mass transport and the evaporation are intrinsically limited by energy constraints. Higher rates of mass transfer lead to increased conductive heat loss, whereas too low rates inhibit evaporation. Thus, water transport is tightly coupled with both evaporation and heat localization.^71,75^

### 3.2 Polymer–water coupling interactions during solar evaporation

On the microscopic scale, water trapped inside hydrogels exists in multiple phases of free water, bound water, and intermediate water, characterized by distinct interaction strength with polymer chains.^72^ These different phases play a significant role in the water evaporation mechanism under solar irradiation. Free water behaves like pure water and is minimally affected by the polymer network. On the other hand, bound water is tightly bound to the polymer through hydrogen bonds or electrostatic interactions, causing a decrease in its mobility and changes in thermodynamics. The third intermediate water corresponds to partially confined water molecules, where the hydrogen bonding structures change.^73^ Consequently, when these water states are heated by the sun, the concentration and dynamics change as influenced by the temperature gradients and polymer relaxation.^74^ Furthermore, the breaking and making of hydrogen bonds within the polymer network may lead to the formation of metastable structures, which will not reach the thermodynamic conditions of the bulk. This non-equilibrium polymer–water system has been proposed to show a decrease in the apparent enthalpy change for evaporation. How extensive this phenomenon is and what impact this has, however, is not widely agreed upon. Further, the temperature variation from one point to another in the house and in the months leads to non-homogeneous water distribution as induced by solar heating. This heterogeneity, in turn, leads to non-trivial phenomena like local swelling and polymer network deformations, further complicating the evaporation process.^76^ Finally, during evaporation, the polymer chain network serves as a scaffold and offers many channels for the transport of water in addition to the usual diffusion channels. In this regard, evaporation is a non-equilibrium process and is no longer considered as a straightforward extension of classical evaporation theory because of a coupled transport process.^77^

### 3.3 Ion transport mechanisms and rejection behavior

Similar to water, salt ions must be managed properly in order to achieve sustainable operations in a solar desalination device. In hydrogels, ion transportation is mediated by the synergy of diffusion, convective flow, and electrostatic interactions, all of which are dictated by the chemical/structural characteristics of polymer networks.^78^ One key ion management process is the Donnan exclusion effect, which occurs due to the presence of fixed charged ions in the polymer network. These fixed ions establish a potential barrier that repels the same charges but attracts oppositely charged ions,^79^ leading to ion partitioning inside/outside the hydrogel. As such, the effect of ion exclusion lowers the effective salt concentration inside the hydrogel. Other effects related to ions include ion–polymer interaction, where specific functional groups on polymer chains interact with ions directly, altering ion mobilities and distributions inside hydrogels.^80^ As in free solutions, the interactions between ions and polymers depend heavily on the chemistry and topology of polymers as well as the types of ions involved. Therefore, these ion–polymer effects enable selective ion removal, which cannot be achieved using solely physical porous media. Furthermore, ion transport is coupled with water transport, as ions will be pulled towards the evaporation front due to convective forces.^81^ Thus, ions accumulate at the surface where convective flow meets diffusion in the bulk solution. In this process, the salt concentration polarizes and the balance of the two processes dictates the ultimate accumulation of ions.

### 3.4 Salt crystallization dynamics and self-regenerating behavior

**Fig. 5:**
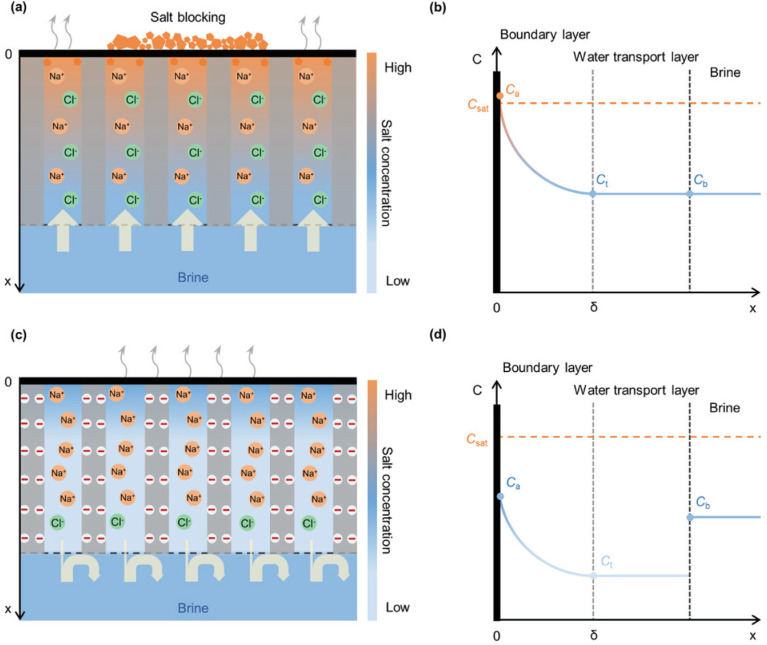
Schematic of the interfacial solar evaporation for brine treatment. (a) Conventional solar evaporators suffering from salt accumulation. (b) Boundary-layer mass-transfer analysis based on salt-ion balance, (*C*_a_ > *C*_sat_), leading to salt crystallization. (c) Salt-resistant solar evaporator. (d) Donnan equilibrium mechanism, in which the confined Na^+^ reduces salt diffusion into the water-transport layer (*C*_t_), maintains *C*_a_ < *C*_sat_, and fundamentally suppresses salt accumulation. Reproduced from ref. 70 with permission from Wiley, copyright 2021.

Salts crystallizing from the water phase pose a serious risk to solar desalination devices since they clog evaporation sites, alter transport pathways, and create mechanical stresses. Compared with traditional evaporators, hydrogels show fundamentally different behaviors in terms of salt crystallization. The site of crystallization either happens at the hydrogel surface or within its bulk, depending on ion concentration, water transport, and nucleation kinetics. Crystallizations preferentially happen at the surface of hydrogel since the salt can be flushed out from the evaporation site,^82^ allowing transport pathways to remain unobstructed. Otherwise, salt crystallization occurs in the bulk and impedes further evaporation by disrupting the polymer network and changing the transport pathway topology. The inherent characteristics of hydrogels give rise to unique advantages in managing the crystallization behavior. First, the high water content of hydrogels facilitates partial dissolution of salt crystals due to continuous hydration. Second, the elasticity of hydrogels allows crystallization to occur without causing catastrophic damage to the system. Lastly, controlling the water and ion fluxes allows a certain amount of self-regulating behavior where the salt continuously gets dissolved. Possible ways to optimize self-regulation include introducing asymmetrical structures in the hydrogel that allow directional water transport.^83^ Alternatively, stimuli-responsive hydrogels can be designed to periodically regulate salt concentrations through periodic hydration and temperature alteration, thereby maintaining a clean evaporation surface.^84^Fig. 5 provides a schematic overview of salt accumulation in conventional solar evaporators caused by concentration polarization during interfacial evaporation, along with a boundary-layer mass-transfer analysis based on the salt-ion balance within the boundary layer. Overall, hydrogels employed in solar desalination systems operate in a highly non-equilibrium environment. The hierarchical structure of hydrogels gives rise to complex phenomena beyond the classical models for understanding water evaporation, highlighting the necessity for a materials-based study. From this perspective, key parameters, such as the network topology and chemical structure of the polymer, are critical to understanding the underlying mechanisms, guiding further development and design.

## 4. Hydrogel architectures for solar desalination

### 4.1 Two-dimensional interfacial evaporators

Layered two-dimensional (2D) interfacial evaporators are a commonly used design for solar desalination based on hydrogels because of their capability to concentrate heat on the air–water interface while reducing the loss of heat energy in the liquid phase. In these evaporators, evaporation occurs primarily at the thin surface layer of the material to be evaporated, and thus, heat-to-vapor conversion is more efficient. The successful implementation of this function is done through the application of layered hydrogel structures, typically composed of a photothermal top layer for absorbing solar radiation, a hydrogel layer as a water transfer medium and for water storage and a bottom insulating/support layer for heat insulation.^85^ Moreover, 2D layered design allows you to decouple different functions in separate layers and optimize both heat and mass transfer processes. Composite hydrogels, featuring the incorporation of light-absorbing additives, such as various carbon nanomaterials, metal nanoparticles, and semiconductors, into the hydrogel polymer matrix are further improvements of this approach for increasing the evaporation efficiency. Such composite hydrogels can perform all the three above-mentioned functions: photothermal energy harvesting, capillary water transportation, and structural stability.^86^ Furthermore, the porosity control in hydrogel materials and their variable wettability allow for water replenishing of the surface and preventing the formation of salt crust thereon. Therefore, composite 2D hydrogels can be considered as efficient tools in achieving a high-performance level of solar desalination due to effective heat localization, water supply, and salt management.^91^

**Fig. 6:**
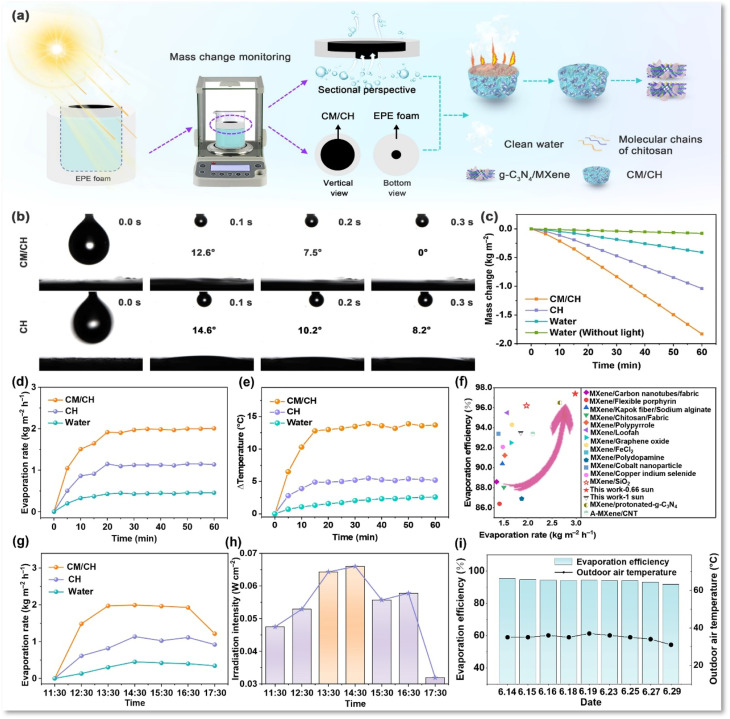
Performance of the solar-powered photothermal evaporation of the hydrogel evaporation system. (a) Experimental setup used to investigate the solar-powered vapor production. (b) Water contact angle measurements for the CM/CH and CH hydrogels. Comparison between the (c) water loss rate, (d) evaporation rate, and (e) surface temperature of CM/CH, CH, and water exposed to natural sunlight (0.066 W cm^−2^). (f) Efficiency of the CM/CH evaporators in comparison with other MXene-based evaporators. (g) Daily evaporation rates for CM/CH, CH, and water. (h) Variations in solar power throughout the day. (i) Effect of outdoor temperature on the efficiency of CM/CH evaporation. Reproduced from ref. 87 with permission from Elsevier, copyright 2026.

Hao *et al.*^87^ prepared a dual-functional photo-responsive hydrogel based on chitosan incorporating g-C_3_N_4_/MXene heterostructures (g-C_3_N_4_/MXene@Chitosan; CM/CH). Light-induced heat generation and ROS (reactive oxygen species) generation along with antimicrobial chitosan activity allow for the fast and efficient elimination of bacteria during water purification process. Many functional groups in chitosan hydrogels form H-bonding interactions with water molecules, leading to water transport facilitation and a decrease in evaporation enthalpy.^89^ As a result, the CM/CH hydrogel demonstrates a water evaporation rate of 2.98 kg m^−2^ h^−1^ and a solar-to-vapor conversion efficiency of 97.4% under irradiation of 1 sun. The evaporation rate of the hydrogel reaches 1.97 kg m^−2^ h^−1^ with 96.2% efficiency under natural irradiance of ∼0.066 W cm^−2^. Due to its antifoul and robust nature, the evaporation efficiency of this hydrogel evaporator is over 91% after 15 days exposure to sunlight outdoors. The system is further capable of treating multi-contaminated water, providing desalination, elimination of heavy metal ions, and decomposition of organic dyes. Thus, such hydrogel can be considered a prospective solar-powered water purification device.^88^Fig. 6 presents the characterization of the performance of the hydrogel evaporator. The schematics of the experiments conducted are illustrated in Fig. 6(a). Fig. 6(b) shows the water contact angle of the CM/CH and CH hydrogels. Fig. 6(c)–(e) compare the amount of water, evaporation rates, and temperature profiles of the CM/CH evaporator, CH evaporator, and pure water under natural sunlight illumination. Fig. 6(f) demonstrates the comparison of the CM/CH hydrogel with other MXene-based hydrogel evaporators. Finally, time dependence on evaporation rate and solar irradiance intensity measured in the course of the day is provided in Fig. 6(g) and (h).

**Fig. 7:**
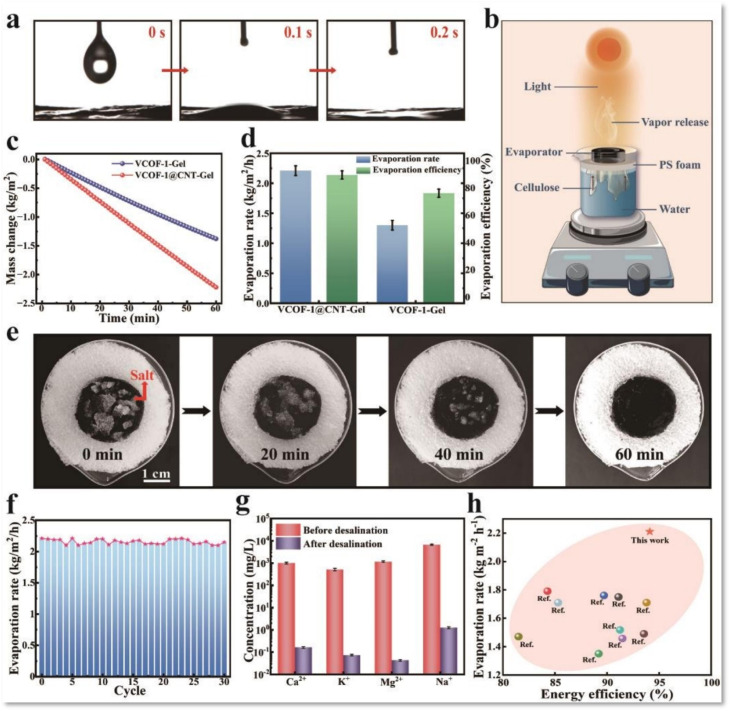
(a) Water contact angle images of the VCOF-1@CNT-Gel interface. (b) Schematic of the designed evaporation setup. (c) Mass loss of various evaporator systems under one sun illumination intensity. (d) Evaporation rate and efficiency of VCOF-1-Gel and VCOF-1@CNT-Gel (*n* = 3 and error bar representing the standard deviation). (e) Salt dissolution images on the VCOF-1@CNT-Gel surface. (f) Cycling solar evaporation process of the evaporator system at 3.5 wt% salinity concentration in 8 hours per cycle. (g) Metal ion concentration before and after the desalination process (*n* = 3 and standard deviation). (h) Evaporation rate and efficiency comparison with other published evaporator devices. Reproduced from ref. 90 with permission from Elsevier, copyright 2026.

Qiuyi Bao *et al.*^90^ developed a covalent cross-linked, multifunctional VCOF-1@CNT-Gel hydrogel by using a vinyl-functionalized covalent organic framework composite (VCOF-1@CNT) as a covalent crosslinker. As illustrated in Fig. 7, VCOF-1@CNT was initially synthesized *via* a reversible hydrothermal imine condensation reaction between 2,5-divinylterephthalaldehyde (DTA), trimethyl-1,3,5-triazine (TMTA), and amine-functionalized carbon nanotubes (NH_2_-CNTs). The resultant composite was then incorporated into a hydrogel structure through the process of radical polymerization, resulting in the formation of strong covalent bonds between VCOF-1@CNT and polyacrylic acid (PAA) polymers.^92^ The combination of strong covalent framework and designed functionalities of the VCOF-1@CNT component allows the resultant hydrogel to possess dual capabilities, *i.e.*, high electromechanical stability for wearable human activity tracking applications and improved water purification efficiency under solar irradiation. The performance characteristics of VCOF-1@CNT-Gel in solar evaporation process are summarized in Fig. 7. Fig. 7(a) illustrates the contact angle measurements demonstrating hydrophilicity of the hydrogel surface. Fig. 7(b) demonstrates the schematic diagram of the evaporation system featuring a two-dimensional path design for water vapor transport. Mass change among different evaporators under 1 sun irradiation is provided in Fig. 7(c). Fig. 7(d) compares the evaporation rate and energy conversion efficiency for VCOF-1-Gel and VCOF-1@CNT-Gel (*n* = 3). Fig. 7(e) illustrates salt dissolution images taken from the surface of the hydrogel, indicating the antifouling property of the material. Fig. 7(f) presents the cyclic evaporation performance of the material tested under 8 hours cycles with 3.5 wt% saltwater. The removal efficiency of different metal ions during the evaporation process was quantified, as shown in Fig. 7(g). The evaporation performance and energy conversion efficiency of VCOF-1@CNT were determined.

**Fig. 8:**
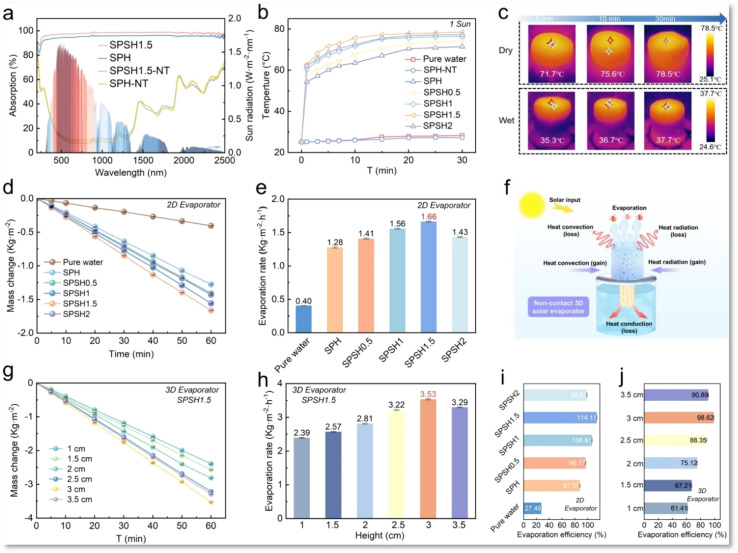
(a) UV–vis–NIR absorption spectra of the wetted SPSH samples based on the AM 1.5 solar spectrum. (b) Variation in the surface temperature with respect to dry and wet SPSH samples between 0 and 30 min. (c) IR images of dry/wet SPSH1.5 after 5, 10, and 30 min of exposure. (d) Mass changes and (e) evaporation rates for the SPSH-2D evaporation system. (f) Noncontact design concept of the three-dimensional SPSH evaporation structure. (g) Mass changes and (h) evaporation rates for SPSH-3D at different height exposure times. Evaporation efficiencies of (i) SPSH-2D and (j) SPSH-3D. Reproduced from ref. 93 with permission from Elsevier, copyright 2026.

A freeze-drying free hierarchically porous hydrogel evaporator (SPSH) based on sodium alginate (SA), poly(vinyl alcohol) (PVA), and sulfonated cellulose nanocrystals (SCNCs)/carboxylated carbon nanotubes (CNTs–COOH) was fabricated by Xiang Hu *et al.*^93^ using an effective mechanical foaming approach. The dual crosslinked SA-PVA matrix along with broadband light scattering induced by SCNCs/CNTs creates a micro-nano framework, which together improves light harvesting, water transportation, and vapor releasing capability.^95^ The functional groups contained in the polymer matrix regulate the state of water molecules, and consequently, lower the equivalent evaporation enthalpy down to 1177.5 J g^−1^ while providing a high evaporation rate of 3.53 kg m^−2^ h^−1^ with 98.5% energy conversion efficiency under 1 sun exposure conditions. In particular, the SPSH1.5 evaporator performs steadily for at least 8 hours in the case of 20 wt% brine without any surface salt crystal formation, and it efficiently degrades organic dyes within the wide pH range.^96^ Seven-day field tests involving three types of water sources, namely, the seawater from the Bohai sea and two different brine samples from southern Xinjiang, China, provided a constant productivity ranging from 9.85 to 14.25 kg m^−2^ day with excellent water quality meeting the WHO standard and being suitable for wheat seedling germination. Notably, the fabrication cost of the presented technology is very low with the figure of $6.45/m^−2^, and the energy-saving mechanical foaming approach leads to a decrease in the carbon dioxide emission by more than 98% compared to traditional freeze-drying processes, making the SPSH hydrogel a promising candidate for large-scale and environmentally friendly production of freshwater from saline sources.^94,97^Fig. 8 evaluates the photothermal properties and evaporating efficiency of SPSH under 1 sun irradiation. Fig. 8(a) illustrates the UV-Vis-NIR absorption spectra of the hydrated SPSH hydrogel compared to the AM 1.5 solar spectrum. Fig. 8(b) shows the variation of the top surface temperature for dried and hydrated SPSH evaporators during the first 30 min. Fig. 8(c) demonstrates the infrared thermal images of dried and hydrated SPSH1.5 evaporators at 5, 10, and 30 minutes. Fig. 8(d) and (e) demonstrate the mass change and evaporation rate of the 2D SPSH evaporator in 60 min. Fig. 8(f) presents the schematics of 3D SPSH evaporators. Fig. 8(g) and (h) provide mass change and evaporation rate of the 3D evaporator with different exposure heights. Fig. 8(i) and (j) illustrate the evaporation efficiencies of 2D and 3D structures.^98^

### 4.2 Polymer-based hydrogel systems

Polymer-based hydrogels have emerged as the principal material matrix for solar-powered interfacial desalination, due to the inherent property of having a crosslinked structure, allowing for the simultaneous regulation of water transport, evaporation, and salt rejection.^99^ Generally, hydrogels prepared using synthetic polymers like polyacrylamide (PAAm), polyvinyl alcohol (PVA), and polyacrylic acid (PAA) exhibit excellent water retention capability without losing the structural stability, thus guaranteeing sufficient water provision to the evaporation site regardless of external pumping. Properties including porosity, hydrophilicity, and transport pathways of hydrogels can be easily controlled by tuning the crosslinking density, functional group, and network structure.^100^ Functionalized and polyelectrolyte hydrogels possess chargeable sites for interaction with ions that lead to salt mitigation mechanisms such as Donnan exclusion and ion redistribution. Hydrogels can also be combined with photothermal materials to create composite structures with excellent light absorption, heat localization, and mass transport properties within a single framework. The structural versatility of hydrogels enables facile manufacturing into films, pores, or anisotropic channels with flexibility in swelling, dehydration, and crystallization-induced stress.^101^ However, the key challenges in fabricating an ideal hydrogel for solar desalination include balancing mechanical strength and high-water content along with chemical/photothermal stability under harsh operating conditions.

**Fig. 9:**
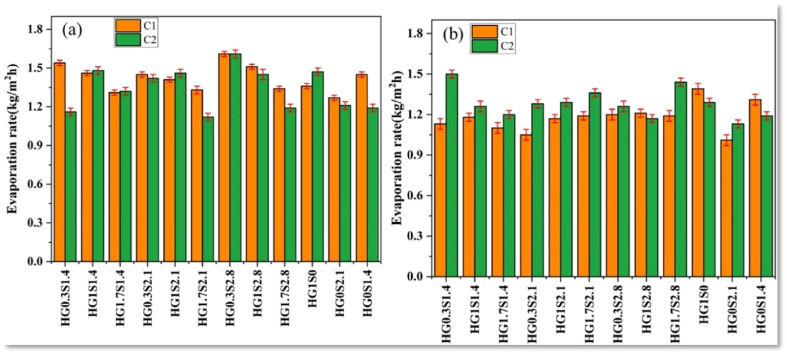
Normalized evaporation rates of hydrogels for one-sun illumination obtained under natural sunlight exposure over two cycles (C1 and C2) for (a) distilled water and (b) saline water. Reproduced from ref. 101 with permission from Elsevier, copyright 2026.

Mitra Tavakoli *et al.*^101^ reported a new type of photothermal hydrogel system made from PVA, PAM, graphene oxide (GO), and silica aerogel (SA) *via* electron beam (e-beam) irradiation in a completely green process, as opposed to traditional chemical crosslinking with hazardous chemicals. Extensive characterizations using FTIR spectroscopy, SEM, EDS, contact angle measurement, swelling test, and compressive strength analyses demonstrated a mechanically durable, anisotropic, and uniform nanomaterial-distributed hydrogel with asymmetric wettability suitable for efficient solar evaporation. Response surface methodology (RSM) was used for optimizing the concentrations of GO and SA, resulting in the optimum value at 0.3% (w/v) of GO and 1.4% (w/v) of SA. At the optimized parameters, the evaporation rate for distilled water and saline water reached 1.54 ± 0.02 kg m^−2^ h^1^ and 1.50 ± 0.03 kg m^−2^ h^−1^, respectively, under natural sunlight.^103^ In this composite, the addition of GO leads to broadband photothermal efficiency and increased mechanical robustness, whereas SA provides buoyancy and reduces heat conduction to the subsurface region.^102,104^ These beneficial characteristics sustain effective surface heating, high water uptake, and stable evaporation. Therefore, we present a promising lightweight and efficient hydrogel platform for sustainable solar desalination. Fig. 9 shows the evaporation rates of the hydrogel samples normalized under natural sunlight in two consecutive cycles (C1 and C2) for (a) distilled water and (b) saline water systems.^102^

**Fig. 10:**
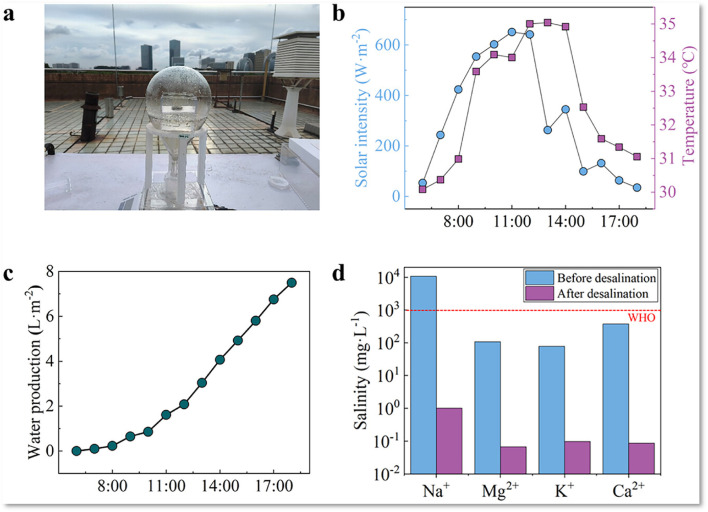
Outdoor solar desalination performance. (a) Picture of the bench-scale setup utilizing the PDAppy-S1.5 hydrogel evaporator. (b) Hourly data on the solar irradiation intensity and air temperature (6:00–18:00). (c) Hourly production rate of freshwater (6:00–18:00). (d) Concentration levels of the four main ions in seawater and the recovered water. Reproduced from ref. 105 with permission from Wiley, copyright 2026.

A polyampholyte hydrogel evaporator was successfully fabricated by incorporating Na_2_SO_4_ in the copolymerization process of AMPS-Na and DMC to create a salting-out effect, resulting in a convenient and scalable solvent-free fabrication strategy. It is worth noting that the presence of Na_2_SO_4_ led to an interwoven porous structure of hydrogel, contributing significantly to enhanced water transportation dynamics. Concurrently, the coexistence of anionic and cationic moieties within the hydrogel enabled pronounced salting-in behavior when exposed to seawater, effectively preventing any salt accumulation at the evaporative interface, thus ensuring sustained performance over time.^105,107^ Consequently, a highly efficient and robust hydrogel evaporator was obtained exhibiting a water evaporation rate of 2.18 kg m^−2^ h^−1^ in real seawater over a period of seven consecutive days without any loss in efficiency. In addition,^106^ the demonstrated robustness and sustainability of the fabricated evaporator under challenging weather conditions proved its applicability on an industrial scale. Hence, this work provides a feasible design for future-generation hydrogel-based solar evaporation technologies.^108^Fig. 10 shows experimental results regarding outdoor desalination performance of the fabricated PDAppy-S1.5 hydrogel evaporator. As illustrated in Fig. 10(a), the photograph depicts the experimental evaporation system used in the experiments. Fig. 10(b) represents hourly data related to the solar radiation intensity and ambient air temperature between 06:00 and 18:00. The hourly freshwater yield obtained during this period is represented in Fig. 10(c). Finally, Fig. 10(d) reports on the concentrations of four essential ions (Na^+^, K^+^, Ca^2+^, and Mg^2+^) present in natural seawater and the distilled freshwater, respectively.^105^

**Fig. 11:**
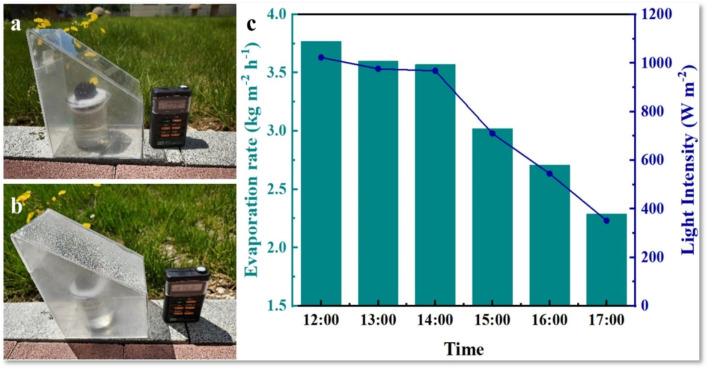
(a and b) Digital photos of the solar distillation system. (c) Evaporation rates along with real-time data on solar irradiation. Reproduced from ref. 109 with permission from Elsevier, copyright 2026.

A sponge-like composite hydrogel interfacial evaporator made up of a polyacrylamide (PAM) hydrophilic matrix and a polyaniline (PANI) photothermal component was introduced in Huang *et al.*’s study.^109^ Such composite material is synthesized in a sequential manner: through Conon solvency photopolymerization of PAM followed by *in situ* oxidative polymerization of PANI. In this case, the Conon solvency effect provides important architectural contribution that changes the internal pore configuration of the PAM hydrogel into a porous open sponge-like one with continuous microchannels, which greatly increases water flow inside the hydrogel matrix.^110,111^ Utilizing this advantage combined with the high photothermal properties of the PANI component, the optimized PAM/PANI hydrogel evaporator reaches a record-high solar evaporation rate of 2.15 kg m^−2^ h^−1^ with 96% energy efficiency, while demonstrating strong salinity resistance and long-lasting operability. Furthermore, to improve the performance of such materials even more, a combination of Conon solvency photopolymerization technology and digital light processing (DLP) 3D printing resulted in the fabrication of the PAM/PANI evaporator with conical surface array architecture. This three-dimensional structure significantly increased the evaporation surface area and enhanced the utilization of sunlight through multi-reflection effects, resulting in an increase in the evaporation rate to 3.73 kg m^−2^ h^−1^, while maintaining the salt resistance and durability properties of the material.^112^ Thus, such 3D-PAM/PANI hydrogel evaporator proved its practical applicability for seawater desalination and wastewaters purification operations in both laboratory and outdoor settings, thereby qualifying as a very promising platform for the sustainable and long-lasting treatment of high-salinity solutions.^113^Fig. 11 demonstrates the outdoor solar desalination performance of a set of 16 cones of 3D-PAM/PANI evaporators.

### 4.3 Bio-inspired and adaptive architectures

Adaptive and bio-inspired hydrogel architecture can be used to achieve a higher efficiency in the field of solar desalination using biological inspirations related to water transfer and regulation in nature. Bio-inspired designs are those inspired by plants, such as the xylem vessels and leaf transpiration system, where the designs contain aligned channels, hierarchies of porous networks, and gradients to allow for directional water transport and evaporation. For example, mangrove-inspired hydrogels can mimic the exclusion of salt naturally in plants by facilitating selective ion transport and blocking salt accumulation at the evaporating surfaces.^114^ Apart from the bio-inspired architecture, the adaptive hydrogel architecture allows the dynamic control of the hydrogel's physical property changes through external stimuli like light, temperature, and salinity to control their swelling and water transport pathways.^115^ Additionally, using structural approaches such as kirigami and origami patterning allows adaptation in light absorption and the evaporating surface area. These adaptive hydrogel materials provide an opportunity for designing advanced, robust, and high-efficiency solar desalination hydrogels.^116^

To solve both the issue of the emergence of green tides on Chinese coasts and freshwater scarcity, Jijin Wu *et al.*^116^ reported a novel method of designing a renewable, sustainable, and low-cost hydrogel solar evaporator from polysaccharides isolated from the green tide algae *Enteromorpha prolifera* (*E. prolifera*). As opposed to other methods such as dumping the algal waste into landfills, which leads to wasted biomass and pollution of the environment, the researchers created solar evaporation materials out of the green algae. The designed hydrogel evaporator exhibits excellent broadband solar absorption (91%), strong hydrophilicity, and anti-salt precipitation properties. Under 1 sun illumination, the hydrogel exhibits an evaporation rate of 1.53 kg m^−2^ h^−1^ with consistent performance throughout 15 evaporation cycles without degradation. Moreover, the stability of the hydrogel has been evidenced by 8 h uninterrupted seawater evaporation under the same solar illumination. Outdoor desalination tests show 99% ionic contaminant removal for K^+^, Ca^2+^, Na^+^, and Mg^2+^ ions in seawater. This is achieved by the recycling nature of the hydrogel.^119^ Therefore, this research presents a sustainable loop approach to dealing with the issues of *E. prolifera* blooming and the pollution of biomass, and at the same time, produce fresh water.^117,120^

**Fig. 12:**
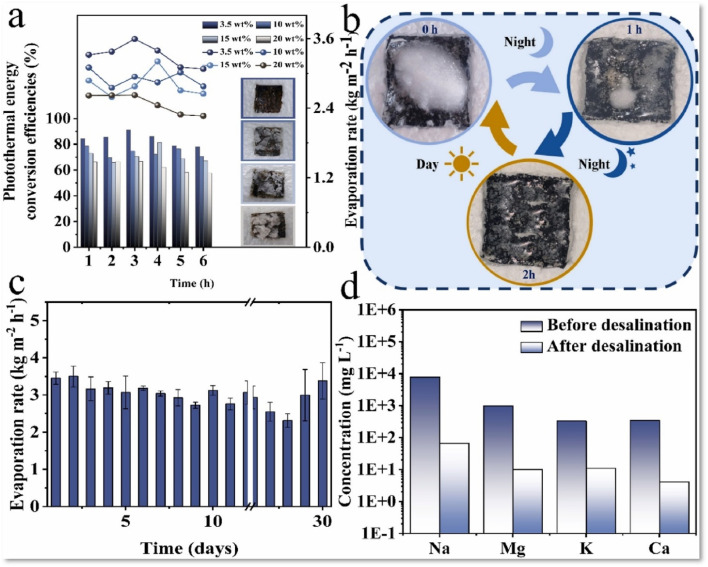
(a) Evaporation rates of CNU@PMI-balsa based on different salt concentrations. Insets: corresponding evaporation images after 6 h. (b) Desalination performances of CNU@PMI-balsa. (c) Evaporation rates of CNU@PMI-balsa in natural seawater within a period of 30 days. (d) Desalination performances of CNU@PMI-balsa in natural seawater. Reproduced from ref. 117 with permission from Elsevier, copyright 2026.

The construction of a high-performance solar evaporator based on the use of hydrogen bond-enhanced supramolecular hydrogel, namely, polyethylene glycol–imidazolidinyl urea, (PMI), along with balsa wood fiber as a natural substrate and NaOH/urea-functionalized carbon nanotubes (CNU), is described by Yongxin Guo *et al.*^117^ In such a combination of materials, the hydrophilic nature of amino groups (–NH_2_) of the PMI polymer, in addition to hydrogen bonding and increased hydrophilicity of the hydrogel matrix due to carbon nanotubes, enables efficient water transportation and reduction of the evaporation enthalpy, which is essential for high evaporation performance of the device. The proposed CNU@PMI-balsa evaporator demonstrated a record-breaking evaporation rate of 3.45 ± 0.08 kg m^−2^ h^−1^ with an irradiance of one sun with more than 95% removal efficiency of salt ions, organic dyes, antibiotics, and herbicides, thus enabling its application not only for seawater desalination but also wastewater treatment.^119^ Stability tests revealed an evaporation rate of 33.77 kg m^−2^ d^−1^ and 32.47 kg m^−2^ d^−1^ of seawater and wastewater, respectively, under 30 days of continuous operation outdoors. Cost-effectiveness evaluation using techno-economic analysis (TEA) and life cycle assessment (LCA) confirmed the competitiveness of the designed technology and offered guidelines for its synthesis optimization. Such a work reveals promising possibilities for water purification by combining natural substrates with advanced materials in the form of supramolecular matrices.^118,120^Fig. 12 demonstrates the performance of seawater desalination by the CNU@PMI-balsa evaporator. Fig. 12(a) provides the evaporation rates for various salt concentrations, with photographs taken of the desalinated sample at the 6 hours point. Fig. 12(b) demonstrates the results of desalination testing under laboratory-controlled conditions. Fig. 12(c) and (d) demonstrate long-term (30 days) results of evaporation and desalination, respectively, when operated continuously in natural seawater.

**Fig. 13:**
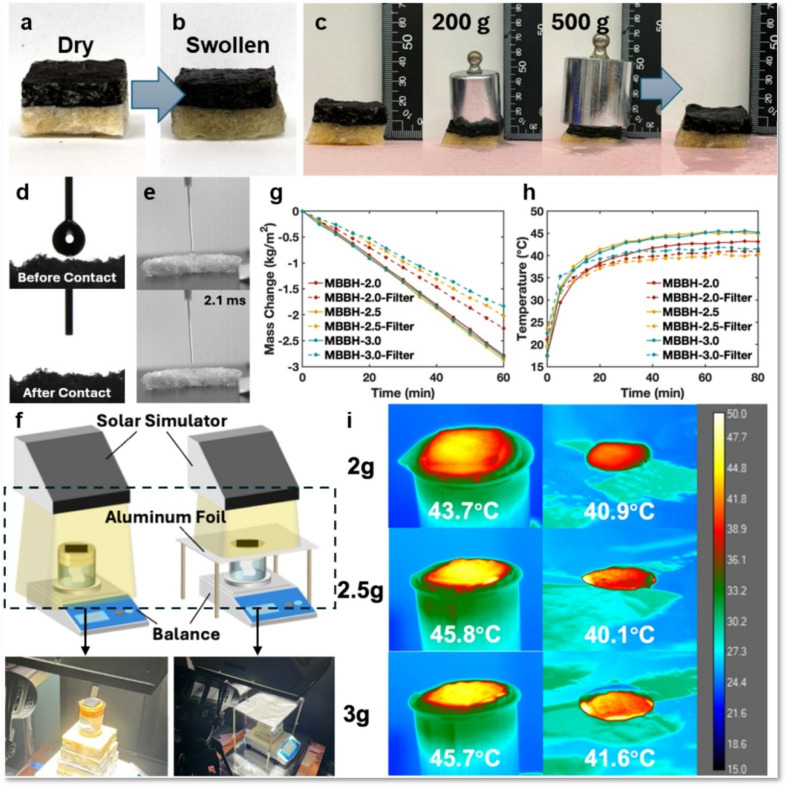
Properties of MBBH, including the mechanical, wettability, and evaporation characteristics. Pictures of MBBH-2.0 when (a) dry and (b) swollen, (c) Compressive stability of swollen MBBH-2.0 in 200 g and 500 g loads. (d) Contact angles of MBBH-2.0. (e) Pictures highlighting the water uptake process. (f) Diagram showing the experimental setup used for solar evaporation, including direct sunlight exposure and filtered light. (g) Mass changes, (h) surface temperatures and (i) evaporation rates of MBBH-2.0, −2.5, and −3.0. Reproduced from ref. 120 with permission from Elsevier, copyright 2026.

Hanqing Liu *et al.*^120^ developed a marine-biomass bilayer hydrogel evaporator (MBBH) using solely marine-derived biomaterials such as chitosan, sodium alginate, and squid ink pigment as a sustainable approach for solar-driven water purification. The bilayer structure is composed of a photothermal black top layer for broadband solar energy harvesting with a white bottom layer, facilitating water transportation and preventing heat loss. A double lyophilization process produces a hierarchically porous network structure, enabling fast capillary water supply, effective solar-to-heat conversion, and superior mechanical properties. Under the irradiation of one sun, the MBBH has an evaporation rate of 2.9 kg m^−2^ h^−1^, which is significantly enhanced to 16.3 kg m^−2^ h^−1^ under concentrated irradiation of five suns without causing damage. Moreover, MBBH can operate in highly salinized solutions with 10 wt% NaCl concentration without significant changes in its performance. By comparing different combinations of water supply strategies and thermal insulation materials, the most efficient design is to feed water indirectly and use a side foam material for thermal insulation.^121,122^ In Fig. 13(a) and (b), the photographs of MBBH-2.0 can be seen in dry and swollen states, indicating superior stability and interfacial bonding properties. In Fig. 13(c), the compressive strength of swollen MBBH-2.0 with 200 g and 500 g weight is demonstrated. Contact angles measured during dynamic wetting are presented in Fig. 13(d). Rapid water absorption was captured in high-speed photography in Fig. 13(e). Finally, in Fig. 13(f), schematic drawings and setup configurations for two irradiations (direct and filtered) are provided. Comparisons among mass changes, surface temperatures, and evaporation rates of three samples (MBBH-2.0, MBBH-2.5, and MBBH-3.0) are shown in Fig. 13(g)–(i)), respectively. The alginate concentration has minor effects on the evaporation rate and temperature under the direct irradiation condition (∼2.9 kg m^−2^ h^−1^, ∼45 °C). Under light filtering conditions, evaporation rate drops to 81%, 71%, and 65% and the surface temperature decreases to ∼41 °C. Table 3 summarizes the hydrogel-based materials for solar-driven desalination.^123,124^

**Table 3:** Summary of hydrogel-based materials for solar-driven desalination

Material system	Fabrication method	Photothermal component	Key functional features	Evaporation rate (kg m^−2^ h^−1^)	Solar-to-vapor efficiency (%)	Solar irradiance	Contaminant removal	Long-term stability	Special features	Ref.
g-C_3_N_4_/MXene@chitosan hybrid hydrogel	Zn^2+^-mediated ionic crosslinking	g-C_3_N_4_/MXene heterostructure	Photothermal + photodynamic (ROS); intrinsic antimicrobial chitosan; H-bonded water networks; and reduced evaporation enthalpy	2.98 (1 sun sim.) and 1.97 (natural sun)	97.4 (1 sun) and 96.2 (natural)	1 sun/∼0.066 W cm^−2^	Salt ions, heavy metals, and organic dyes; bacterial eradication	>91% after 15 days outdoor	Dual-mode light response; biofouling resistance; and multi-contaminant treatment	125
*Enteromorpha prolifera* polysaccharide hydrogel	Polysaccharide extraction + hydrogel formation from algal biomass	*E. prolifera* pigment/biomass	High solar absorption (∼91%); excellent hydrophilicity; suppressed salt deposition; and recyclable evaporator	1.53	—	1 sun	K^+^, Ca^2+^, Na^+^, and Mg^2+^ (up to 99% removal)	Stable over 15 cycles and 8 h continuous seawater evaporation	Valorization of algal waste; eco-friendly and cost-effective; and closed-loop strategy	126]
PEG-imidazolidinyl urea/balsa wood/CNT composite	H-bond supramolecular assembly + NaOH/urea CNT functionalization + balsa integration	NaOH/urea-functionalized CNTs (CNU)	H-bond enhanced supramolecular network; –NH_2_ hydrophilicity; efficient water transport; reduced evaporation enthalpy; and TEA- and LCA-validated	3.45 ± 0.08	>95% pollutant removal	1 sun	Salt ions, dyes, antibiotics, and herbicides (>95%)	33.77 kg m^−2^ d^−1^ (seawater, 30 d) and 32.47 kg m^−2^ d^−1^ (wastewater, 30 d)	TEA and LCA cost validation; natural fiber + supramolecular integration	127
PVA/PAM/graphene oxide/silica aerogel composite	Electron-beam (e-beam) crosslinking (additive-free and clean process)	Graphene oxide (GO)	Asymmetric wettability gradient; anisotropic structure; SA provides buoyancy and limits heat loss; and RSM-optimized GO and SA concentrations	1.54 ± 0.02 (distilled) and 1.50 ± 0.03 (saline)	—	1 sun (ambient sunlight)	Saline water desalination	Stable over 2 cycles; high water uptake	Green e-beam crosslinking; scalable and additive-free; and energy-limited deployment	128
Marine-biomass bilayer hydrogel (chitosan/sodium alginate/squid ink)	Double lyophilization → hierarchical porous network	Squid ink pigment (black layer)	Bilayer design: photothermal black layer + insulating white support; rapid capillary water supply; and indirect water feeding + side-foam insulation	2.9 (1 sun) and 16.3 (5 sun)	—	1 sun/5 sun	Salt resistance up to 10 wt% NaCl	Stable under concentrated illumination; no structural degradation	Fully marine-sourced biopolymers; scalable and low-cost; and bilayer thermal management	129
Polyampholyte hydrogel (AMPS-Na/DMC copolymer)	Na_2_SO_4_-induced salting-out copolymerization (solvent-free)	Polypyrrole (doping-assisted)	Interconnected porous structure *via* salting-out; anionic/cationic units for salting-in behavior; and stable evaporation interface	∼2.18 (real seawater)	—	1 sun	Na^+^, K^+^, Ca^2+^, and Mg^2+^ ion removal	Stable over 7 days; robust under harsh conditions	Scalable solvent-free synthesis; salt-responsive swelling; and bioinspired polyampholyte design	130
VCOF-1@CNT/polyacrylic acid covalent hydrogel	Hydrothermal imine condensation + radical polymerization (covalent crosslinking)	Carbon nanotubes (CNT) in COF composite	Covalent COF crosslinks for structural robustness; 2D water path design; anti-salt fouling surface; and dual function: evaporation + wearable sensing	—	—	1 sun	Salt ions (metal ion removal > reported benchmarks); organic contaminants	Stable over cyclic 8 h runs at 3.5 wt% NaCl	Dual-function: solar evaporation + human activity monitoring; COF-based covalent network	131
SA-PVA-SCNC/CNT–COOH hydrogel	Mechanical foaming (freeze-drying-free); dual crosslinking	Carboxylated CNTs (CNT–COOH)	Hierarchical porous structure; broadband light scattering (SCNC/CNT); reduced evaporation enthalpy (1177.5 J g^−1^); low cost ($6.45 m^−2^); and 98% lower carbon emissions *vs.* freeze-drying	3.53 (lab, 1 sun) and 9.85–14.25 kg m^−2^ d^−1^ (outdoor, 7 d)	98.5	1 sun/outdoor	Organic dyes; extreme pH; and salt ions (WHO-compliant output)	Stable for 8 h in 20 wt% brine; 7 days outdoor (Bohai seawater and Xinjiang brines)	Supports wheat germination; ultra-low carbon footprint; and freeze-drying-free scalable process	132
PAM/PANI sponge-like composite hydrogel (2D- and 3D-printed)	Cononsolvency photopolymerization + *in situ* oxidative polymerization; DLP 3D printing for 3D architecture	Polyaniline (PANI)	Cononsolvency-induced open porous network; conical array 3D surface for expanded lateral evaporation area; multiple light reflections; and excellent salt resistance	2.15 (2D flat) and 3.73 (3D conical)	96 (2D flat)	1 sun/outdoor	Seawater desalination; wastewater purification	Stable long-term; no salt fouling in both 2D and 3D configurations	DLP 3D-printed conical architecture; scalable to high-salinity brine treatment	133

## 5. Challenges, opportunities, and future perspectives

Although significant advancements have been made in hydrogel materials for solar desalination, research on these hydrogels remains in the early stages, and many aspects of this emerging technology have not been fully addressed. Several excellent water evaporation rates have been reported, and some new multi-functional material designs have been suggested, but they are mostly tested in idealized laboratory settings and have limited application potential.^134^ Hence, in addition to material optimizations and improvements, an understanding of the complex phenomena responsible for the performance of hydrogel desalination systems is urgently needed. The first and most important requirement would be to acquire a fundamental understanding of the evaporation behavior in confined, polymeric environments when they are exposed to solar light. The evaporation of bulk water can easily be expressed through classical thermodynamic equations, while the evaporation in the hydrogel system is described by the presence of heterogenous water states, the changing polymer–water interactions, and the high spatiotemporal gradients.^135^ These features give rise to non-equilibrium behaviors that cannot be adequately modeled using conventional theories. One of the problems in question is the decrease in evaporation enthalpy observed for hydrogels. Several hypotheses exist regarding its origin. Some suggest that it is caused by the presence of intermediate and bound water and changed hydrogen bonding networks, whereas others assume experimental errors or the failure to account for some energy fluxes.^136–139^ Therefore, rigorous thermodynamic theories that include the impact of confinement on water properties as well as advanced diagnostics capable of examining them *in situ* are required.^140–144^Table 3 highlights the hydrogel-based materials for solar-driven desalination.

Another problem facing researchers and engineers is that in hydrogels, evaporation is tightly coupled to the transport of water molecules and thermal dissipation. The ability of hydrogels to provide sufficient water for evaporation is associated with an increased water content, which creates additional channels for thermal losses.^145^ Thus, a delicate balance must be maintained between the water supply and heat conservation. To overcome this fundamental limitation, future work should focus on the creation of anisotropic and/or heterogeneous hydrogel structures that would guide water transport while suppressing conductive heat losses.^146^ Other key areas that should be considered involve the modelling of coupled water, heat and ion transport in hydrogel systems. While different theoretical models are currently available, they are used in isolation, but they are highly interrelated. For example, the ion concentration alters the osmotic pressure in the immediate environment, which in turn influences water transport; temperature differences alter the diffusivity; and temperature differences alter the interactions between the polymer and the water. This way, the nonlinear (NLFB) effect comes into the picture that can give rise to unexpected behavior.^147^ However, the use of the improvements in the theoretical description of the water evaporation in hydrogels would require Multiphysics models to account for the transport phenomena from the polymer chain level up to system level. Such theories need to be validated using appropriately designed experimental studies.^148–150^ In addition to fundamental scientific issues, engineering issues are also necessary to allow the use of hydrogels in practice as a desalination device. The high sensitivity of the hydrogel structures because of repeated swelling and shrinking, exposure to solar radiation and harsh conditions provided by the feedwater (salt ions, organics and microorganisms) is a serious threat to their durability.^151,152^ The use of a double-network structure, dynamic crosslinking and the introduction of anti-fouling functions are promising but must be tested in detail to prevent long-term stability problems. Specifically, the effects on hydrogels performance and durability due to the combined effect of salt crystal growth with biofouling and other environmental stresses need to be studied. The other issue here is the field of view or “scale”. Presently, most hydrogels have shown high performance only in small-scale processes and products, which require advanced microstructures and fabrication technologies. As the dimensions of the device are increased, unwanted features such as uneven water distribution, enhanced heat losses and mechanical instabilities appear.^153,154^ It is essential to develop appropriate scaling-up strategies, allowing the precision of hydrogel structures as well as their excellent functional properties and mechanical strength to be maintained. Furthermore, the need for the demand to integrate the hydrogel evaporators with bigger desalination systems must be addressed. Despite all the efforts made to improve the performance of the evaporators, little is known about condensation, water collection and heat management.^155^ In practice, the efficiency and effectiveness of a whole system rather than an evaporator alone are crucial. When moving to system-level design, only optimizing the hydrogels along with the condenser, thermal management method, and operating conditions are necessary. Systems with multiple-stage or hybrid systems, which will allow for latent heat recycling, are promising.^156^

**Fig. 14:**
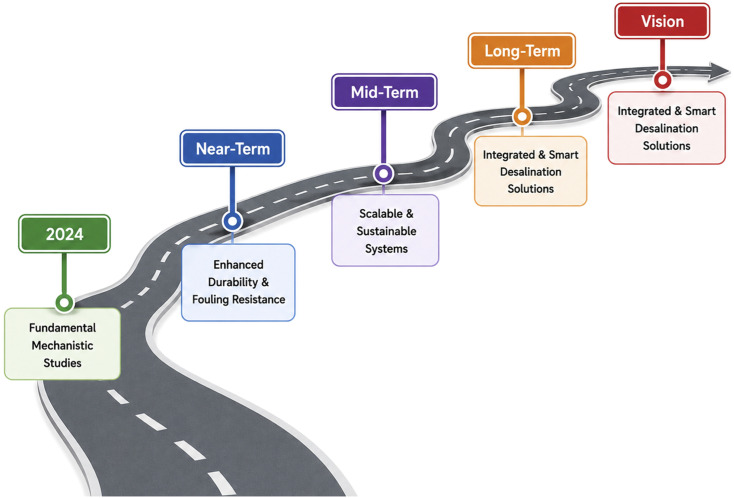
Roadmap illustrating the potential future directions of hydrogel-based solar desalination.

Finally, hydrogel desalination technologies are expected to be more complex and flexible in the future. With the addition of responsive properties of hydrogels, water transport, evaporation and salt retaining properties will be dynamically controlled to: external parameters. Smart hydrogels can provide the basis for creating self-optimizing systems. Simultaneously, the heterogenous combination of hydrogels and aerogels, membranes, or other material systems provides opportunities for new combinations to be created, a chance to further enhance the thermal properties, stability and multi-functionality of devices.^157^ Moreover, developments in data-driven approaches and computational materials science are expected to gain increasing importance. The complexity of hydrogel systems including chemical compositions, network structures, and water transport can be advantageously used to implement artificial intelligence and optimization algorithms.^158^ Overall, the translation of hydrogels for solar desalination applications into a commercial product requires overcoming the challenges associated with performance, durability, scaling, and sustainability. Alongside technological innovations in materials design, it requires a comprehensive approach considering engineering and fundamental science factors. Thus, hydrogel-based solar desalination should be seen not only as a materials science challenge but as a multidisciplinary field encompassing polymer science, transport physics, environmental engineering, and systems design. Continued progress in these related areas is expected to help hydrogel desalination technologies make significant contributions to the provision of fresh water in the years to come.^159,160^Fig. 14 shows a roadmap illustrating the potential future directions of hydrogel-based solar desalination.

## 6. Conclusions

Indeed, the use of hydrogels in solar-powered desalination has become very promising mainly due to the possibility of realizing the integration of water transport, storage, and evaporation processes in one and coherent system. Different from conventional materials, hydrogels control the coupling of water, heat, and ion transport *via* their sophisticated hierarchical structure. The behavior of these materials is determined mostly by the interactions of polymers and water as well as the existence of various types of water, which altogether determine evaporation process. At the same time, water transport and the dynamics of dissolved salts in such systems are controlled by capillary flows, diffusion, and electrostatic interactions, all of which determine their stability during long operation. Thus, an efficient desalination process would require both efficient evaporation and proper salt separation. Significant achievements in developing innovative architectures, from hybrid materials to bioinspired systems, have been made toward improving the performance. However, these advancements still largely rely on the trial-and-error approach and call for the establishment of reliable and predictive relations between structural characteristics, properties, and performance. The implementation of standard testing procedures and consistent performance criteria will be needed to allow proper comparison among different works. It will be crucial for future research to focus on combining innovative materials with detailed knowledge of their behavior and system-level engineering of these processes.

## Author contributions

Tholkappiyan Ramchandran: concept, writing–review and editing, writing – original draft, methodology, investigation; Bathina Chaitanya: formal analysis; Vadivelan Subramaniyan: formal analysis, Ramesh Kumar Raji: reviewing and editing, formal analysis, Shanmugavel Chinnathambi: formal analysis, Ganesan Subramanian, Karthishwaran Kandhan: formal analysis; and Fathalla Hamed, Renuka Seenivasan: formal analysis.

## Conflicts of interest

There are no conflicts to declare.

## Data Availability

No primary research results, software or code have been included and no new data were generated or analysed as part of this review.
